# Dark tone quality and vocal tract shaping in soprano song production: Insights from real-time MRI

**DOI:** 10.1121/10.0005109

**Published:** 2021-07-09

**Authors:** Elisabeth Lynn, Shrikanth S. Narayanan, Adam C. Lammert

**Affiliations:** 1 Department of Biomedical Engineering, Worcester Polytechnic Institute, Worcester, Massachusetts 01690, USA; 2 Signal Analysis and Interpretation Laboratory, University of Southern California, Los Angeles, California 95616, USA erlynn@wpi.edu, shri@ee.usc.edu, alammert@wpi.edu

## Abstract

Tone quality termed “dark” is an aesthetically important property of Western classical voice performance and has been associated with lowered formant frequencies, lowered larynx, and widened pharynx. The present study uses real-time magnetic resonance imaging with synchronous audio recordings to investigate dark tone quality in four professionally trained sopranos with enhanced ecological validity and a relatively complete view of the vocal tract. Findings differ from traditional accounts, indicating that labial narrowing may be the primary driver of dark tone quality across performers, while many other aspects of vocal tract shaping are shown to differ significantly in a performer-specific way.

## 1. Introduction

Dating at least to the work of Manuel Garcia (Garcia, 1840), vocal pedagogy has identified a tone quality termed “dark” as desirable for human voice performance. Darkness is often associated with the additional terms “warm,” “round,” and “covered” and is a key component of the coveted “chiarascuro” (“bright-dark”) tone quality, an aesthetically important property of Western classical voice performance (Stark, 1999). Darkness is associated acoustically with lowered formant frequencies (Sundberg, 1970; Sundberg, 1973) and may stem from specific vocal tract shaping, including a lowered larynx and widening of the pharynx (Bloothooft and Plomp, 1986; Hertegård, 1990). Evidence further suggests that vocal tract shaping counter to that associated with darkness (i.e., larynx raising, pharynx widening) results in bright tone quality commonly described as “twang” (Titze *et al.*, 2003; Perta *et al.*, 2020), which is possibly also accompanied by a wider lip opening and a constricted oral cavity (Story *et al.*, 2001). Darkness is considered complementary, and not opposed, to brightness (despite terminological implication), as one of the two components of chiarascuro. Bright tone quality is associated with prominent high-frequency energy and stems from distinct mechanisms, most notably a tight closure of the vocal folds (Stark, 1999).

Despite what is known about the origins of dark tone quality in vocal tract shaping, technological limitations have made it challenging, until recently, to observe the production of dark tone quality dynamically, during natural voice performance, and with a relatively complete view of the vocal tract. Therefore, the state of knowledge about this aesthetically important tone quality is limited and incomplete, and several open questions remain. First, can it be confirmed that aspects of vocal tract shaping noted by prior studies as being associated with dark tone quality are present in a dynamic, ecologically valid production context? Second, are there other additional aspects of vocal tract shaping, currently unknown, that contribute to the production of dark tone quality? Finally, are the specific aspects of vocal tract shaping observed in association with dark tone quality theoretically predictable on the basis of principles of physical acoustics? The present study aims to address these questions using real-time magnetic resonance imaging (rtMRI) to quantify and analyze vocal tract shaping during voice performance of professionally trained sopranos displaying a range of tone qualities. The use of rtMRI allows for examination of relevant vocal tract shaping across a breadth of postures and in a more natural production context. These advantages facilitate increased ecological validity of experiments, and rtMRI has been used extensively in the study of singing (e.g., Echternach, 2016) and speech (e.g., Narayanan, 2014) for this reason.

## 2. Method

### 2.1 Articulatory analysis

The first and second stated goals of the present study are, respectively, (a) to investigate whether the aspects of vocal tract shaping reported by prior studies as being associated with dark tone quality are present in the multimodal imaging data obtained in a dynamic, ecologically valid production context and (b) to determine whether any additional aspects of vocal tract shaping, currently unknown, contribute to the production of dark tone quality. Both of these objectives were addressed using rtMRI technology to acquire videos, with synchronized audio, of human vocal performance exhibiting dark tone quality and to analyze those data using region of interest (ROI) image processing and subsequent statistical analysis.

#### 2.1.1 Data acquisition

The subjects for the present study were four female native speakers of American English between 22 and 30 years of age (subject identifiers: H5, S2, M1, and L4). All subjects were sopranos trained in the Western classical tradition and at the time of the experiment were graduate students in the University of Southern California (USC) Thornton School of Music Department of Vocal Arts and Opera. Subjects sang portions of “Susanna's aria” from Mozart's Marriage of Figaro. This aria was selected for the present experiment data collection because it was well known to the performers and is widely performed. Subjects were instructed to sing the specified portion in their *normal* performance style (in instructions identified as “ringing”), especially *dark* (identified as “covered”) and especially *non-dark* (identified as “bright”) tone qualities, with separate acquisitions made of each of the three qualities, all at the same tempo and in the key of D major. The primary comparison of interest was between the dark and non-dark styles, with the normal performance style providing a basis for normalizing articulatory and acoustic data from each subject to facilitate comparisons across subjects.

During data collection, the subjects were in a supine position. Sopranos were all accompanied in the experiment by their vocal teacher, who was the individual responsible for communicating the preferred performance style to the sopranos using terminology about which the teacher and soprano had a common understanding from their training. Consistent with the present focus on production, the determination of whether the intended performance outcome was achieved during the experiment was left to the sopranos, and no explicit perceptual assessments were conducted.

**Fig. 1.**
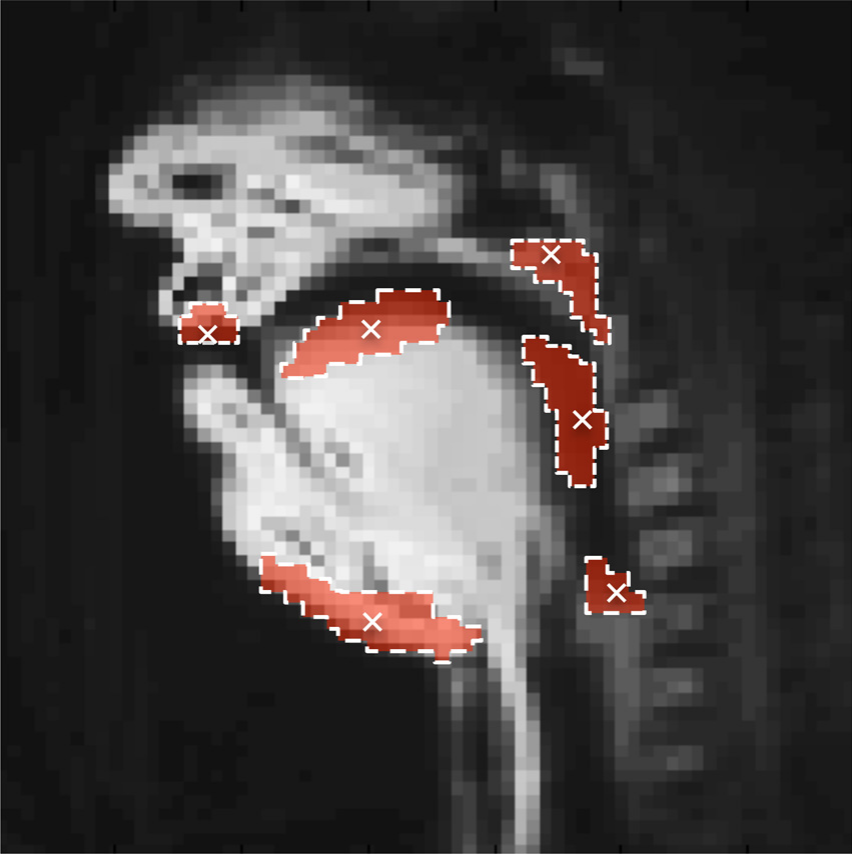
Subject M1 as represented by the mean of all images acquired during her dark (“covered”) aria performance. The six ROIs are superimposed in red, with the selected pixels used to form each region shown as a white “x.” ROIs correspond to labial, coronal, and pharyngeal regions, a region in the larynx immediately superior to the glottis, and regions capturing velum raising and jaw lowering.

**Fig. 2.**
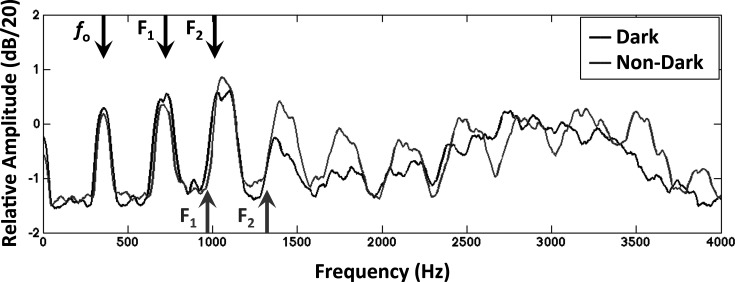
Frequency spectra estimated from audio recordings of subject H5 producing the vowel /a/ in the word “bella.” Spectra from both the dark and non-dark production are shown. Spectra were reconstructed by averaging across windows of a short-time Fourier transform with a 60 ms analysis window, 20% window overlap, Blackman–Harris weighting, and approximately 600 ms total duration. The harmonic structure can be seen, with the spacing between harmonics corresponding to the fundamental frequency (*f*_o_), which was ∼357 Hz in both productions. The first two formant frequencies for each production as computed by praat are also indicated. A broad shift in energy toward lower frequencies can be seen when comparing the dark production to the non-dark one, corresponding to the lower formant frequencies in the dark production.

The rtMRI data were collected on a GE Healthcare (Chicago, IL) Signa 1.5 T scanner, using a 13-interleaf spiral gradient echo pulse sequence with a repetition time (TR) of 6.5 ms. The field of view covered an area of 20 × 20 cm, oriented along the midsagittal plane (see Fig. 1). Images were reconstructed using a sliding-window procedure, creating an image sequence with an effective rate of 22.41 frames/s. Spatial resolution of the reconstructed images was approximately 2.9 × 2.9 mm pixel size in the midsagittal plane (images 68 × 68 pixels in size), with slice thickness of approximately 6 mm. Further details regarding the protocol can be found in Narayanan *et al.* (2004), and an earlier study by Bresch and Narayanan (2010) with the present dataset investigated resonance tuning in soprano singing. Examples are available online (Lynn *et al.*, 2021). Audio was simultaneously recorded at a sampling frequency of 20 kHz using an MRI-safe optical microphone and subsequently denoised according to the protocol described in Bresch *et al.* (2006). Bresch's method promises approximately 30 dB improvement in signal-to-noise ratio using a model-based procedure to suppress the MRI gradient noise. However, this model-based procedure does not account for other noise sources, including background noise caused by the cryogen pump and ventilation system, which are generally expected to be much lower in amplitude than the gradient noise. Representative spectra can be seen in Fig. 2.

All image sequences were cropped to the aria portion corresponding to the lyrics “Deh vieni, non tardar, o gioja bella/Vieni ove amore per goder t'appella.” Cropping was performed by listening to the recorded audio and identifying the frames immediately preceding the audible onset of “Deh” and immediately following the audible offset of “t'apella.” These frames were taken as the first and last frames of the relevant sequence for that recording, and the audio was cropped correspondingly.

#### 2.1.2 Region of interest analysis

A ROI analysis was used to extract articulator-specific kinematic variables during vocal performance in six relevant areas of the midsagittal plane. ROI analysis has been increasingly used as a method of analyzing speech articulation in rtMR images. The method is based on the fact that pixel intensities in rtMR images are largely a function of soft tissue density in the image plane and are therefore closely related to the positions of the speech articulators. Four regions were placed along the length of the vocal tract, with the aim of measuring narrowing of the vocal tract in associated areas. ROI analysis performed in this way has been validated as a method of estimating vocal tract cross-distances from rtMRI data (Lammert *et al.*, 2013b). The selection of regions was informed by the acoustic analysis and physical acoustic simulations—the latter being described in the supplementary material^1^—which indicate that formant frequency changes observed in these subjects were driven by narrowing of the lips and pharynx, combined with widening of the coronal region and of the region of the larynx immediately superior to the glottis (i.e., the laryngeal vestibule, hereafter referred to as *larynx*, for convenience). In addition, a region capturing velum raising was considered, because the velum has sometimes been implicated in tone quality considerations (e.g., in the context of pedagogical references to “head voice” versus “chest voice”), as well as a region capturing movement of the jaw.

The size and shape of the ROIs were determined automatically, for a given image sequence, on the basis of pairwise correlations in pixel intensity fluctuation over time, using the method described by Lammert *et al.* (2010). Region placement was based on manually selected seed pixels, frame-to-frame intensity variations of which are used as a reference for determining membership in the ROI. Seed pixels were located within the anatomically relevant areas of the image plane (i.e., near the lips, pharyx, coronal region, larynx, velum, and jaw) and identified following visual inspection of the vocal tract anatomy, represented by the pixel-wise mean image of the image sequence. A visual representation of the regions used is shown in Fig. 1.

#### 2.1.3 Statistical analysis of articulations

Within-subject, articulator-specific differences associated with dark and non-dark tone quality types were tested by performing two-sample *t* tests on corresponding kinematic variable values in the dark and non-dark conditions. Across-subject, articulator-specific differences associated with tone quality in terms of average vocal tract shape were tested using paired *t* tests on within-subject kinematic variable means. Means for the dark and non-dark conditions were first normalized to a proportion of the “normal” condition mean.

### 2.2 Acoustic analysis

Dark tone quality is associated acoustically with a lowering of formant frequencies (Sundberg, 1970; Sundberg, 1973). The synchronous audio, recorded simultaneously with rtMRI data of the vocal instrument in action, affords the opportunity to confirm that dark tone quality, in this acoustic sense, is exhibited by the subjects in the present study. Moreover, observed differences in formant frequencies found in the present analysis were subsequently used as the basis for theoretical predictions regarding specific changes in vocal tract shape that drive those acoustic differences, described in the supplementary material.^1^ Such predictions can, in turn, be used to inform the regions of interest considered as part of the articulatory analysis of the rtMRI data. Thus, an analysis of formant frequencies across all sonorant sounds was conducted with the intention of assessing overall mean differences in formant frequencies in the different tone quality conditions.

#### 2.2.1 Formant frequency analysis

Audio was analyzed using praat (Boersma, 2001) to perform formant and fundamental frequency tracking. praat was configured to find a total of seven formants in the range 0–5000 Hz, with window length set to 0.025 s and the dynamic range set to 90.0 dB. These settings closely follow those reported in prior work on acoustic analysis of the singing voice (Titze *et al.*, 2017). praat's suggested formants were overlaid on a spectrogram of the audio, to verify a reasonable correspondence between the formants and visible bands of high energy in the signal. The first three formants (i.e., F_1_, F_2_, and F_3_) were taken as the lowest three formants suggested by praat, in terms of frequency, using these settings.

It is expected that, at the high fundamental frequencies observed during singing, formant frequency estimates obtained using praat's linear predictive coding (LPC)-based algorithm may be biased toward nearby harmonics of the fundamental frequency. Several techniques have been developed that allow for formant frequency estimation that is robust to such bias (e.g., Alku *et al.*, 2013; Story and Bunton, 2016). However, the goal of the present acoustic analysis is not to accurately ascertain the values of individual formant frequencies, but rather to ascertain the mean formant frequencies across all voiced segments produced in a given recording and to compare differences between those means across conditions. Accurate estimation of mean formant frequencies is possible despite the expected bias due to statistical properties of the mean as a measure of central tendency. Specifically, for a collection of *i* = 1, 2, 3, …, *n* measured formant values, *F_i_*, each corrupted by additive noise $\varepsilon_{i}$, the mean formant value will be estimated as

$$\mu =\frac{1}{n}\sum_{i=1}^{n}F_{i}+\varepsilon_{i}=\frac{1}{n}\sum_{i=1}^{n}F_{i}+\frac{1}{n}\sum_{i=1}^{n}\varepsilon_{i}=\mu_{F}+\mu_{\varepsilon },$$

where $\mu_{F}$ is the mean uncorrupted formant value, and $\mu_{\varepsilon }$ is the mean of the noise. If one assumes that the values of $\varepsilon_{i}$ are symmetrically distributed—as there is no *a priori* expectation that the bias mentioned above will be toward either a higher or lower harmonic—the value of $\mu_{\varepsilon }$ will tend toward zero as *n* becomes large, meaning that $\mu \approx \mu_{F}$. The value of $\mu_{\varepsilon }$ in general will be less than $\sigma_{\varepsilon }/\sqrt{n}$, where $\sigma_{\varepsilon }$ is the standard deviation (SD) of ε, which can be easily verified using the definition of standard error of the mean.

Fundamental frequency (*f*_o_, called *pitch* within praat) tracking was configured to find values in the range 75–1000 Hz using praat's autocorrelation-based method. The primary purpose of calculating estimates of *f*_o_ was for removing non-sonorant sounds from subsequent analysis. Any formants estimated during analysis frames when a reliable *f*_o_ could not be found by the algorithm during the temporally closest *f*_o_ analysis frame were eliminated from further consideration.

#### 2.2.2 Statistical analysis of acoustics

Within-subject, formant-specific differences associated with dark and non-dark tone quality types were tested by performing one-tailed, two-sample *t* tests on formant frequency values from the dark and non-dark conditions. Across-subject, formant-specific differences associated with tone quality in terms of formant frequencies were tested using paired *t* tests on within-subject formant frequency means. Means for the dark and non-dark conditions were first normalized to a proportion of the “normal” condition mean. All hypothesis tests were one-tailed tests, owing to the prior expectation that formants should lower with dark tone quality.

## 3 Results

**Fig. 3.**
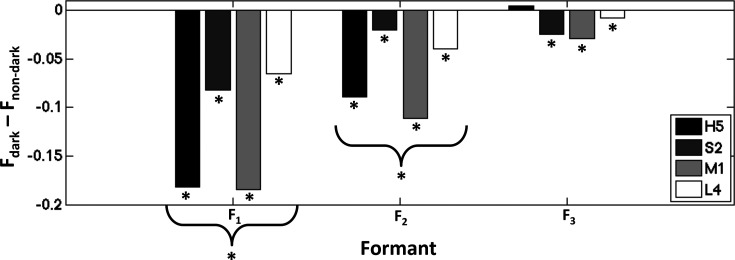
Mean ROI intensity (M) differences between the dark and non-dark performance conditions, expressed as a proportion of mean ROI intensity during the normal performance condition. Differences are shown for each ROI, representing narrowing of the labial, coronal, pharyngeal, and laryngeal regions of the vocal tract. Individual bars represent subject-specific differences. Negative difference values correspond to lower mean formant frequencies during the dark performance. Higher values for the lips, coronal, pharynx, and larynx regions indicate a more constricted vocal tract in that region. Higher values in the velum and jaw regions represent a more raised velum and jaw, respectively. Asterisks (*) indicate corresponding significant effects, described in Sec. 3, within subjects (below/below bars) and across subjects (curly brackets).

Mean differences in ROI intensity, comparing dark and non-dark performance conditions, can be seen in Fig. 3 for each subject. All four sopranos exhibited significant within-subject differences in mean labial narrowing between the dark and non-dark conditions (*p* ≪ 0.001). The majority of sopranos exhibited such within-subject differences in mean coronal narrowing (*p*_S2_ < 0.01; *p*_M1_ ≪ 0.001, *p*_L4_ < 0.001). Two of the four sopranos exhibited such differences in mean pharynx narrowing (*p*_M1_ < 0.01, *p*_L4_ < 0.05). All sopranos exhibited such within-subject differences in mean larynx narrowing (*p*_H5_ < 0.001; *p*_S2_ ≪ 0.001, *p*_M1_ ≪ 0.001, *p*_L4_ ≪ 0.001). The majority of sopranos also exhibited such within-subject differences in mean velum raising (*p*_H5_ ≪ 0.001; *p*_S2_ < 0.001, *p*_L4_ < 0.05). The majority of sopranos exhibited such within-subject differences in mean jaw lowering (*p*_H5_ < 0.01; *p*_S2_ ≪ 0.001, *p*_M1_ ≪ 0.001).

Across subjects (see Table 1), the labial region was found to be consistently narrower during the dark condition versus the non-dark condition, a difference that achieved statistical significance. The larynx region was found to be consistently wider during the dark condition versus the non-dark condition, but this difference did not achieve statistical significance. No consistent or statistically significant differences were observed in mean coronal narrowing, mean pharynx narrowing, mean velum raising, or mean jaw lowering.

**Table 1.** Results of paired *t* tests conducted to determine statistically significant across-subject, articulator-specific differences associated with tone quality in terms of average vocal tract shape. Across-subject means (M) and SDs of within-subject kinematic variable means are shown, along with the degrees of freedom (df), as well as the resulting *t* statistic and *p* value.

Region	Dark		Non-dark		df	*t*	*p*
	M	SD	M	SD			
Lips	0.57	0.43	−0.26	0.08	3	4.31	0.02
Coronal	0.22	0.53	0.38	0.63	3	−1.86	0.16
Pharynx	0.01	0.13	−0.04	0.12	3	1.93	0.15
Larynx	−0.14	0.16	0.09	0.11	3	−2.29	0.11
Velum	0.02	0.06	0.04	0.03	3	−0.87	0.45
Jaw	0.19	0.18	0.12	0.27	3	0.83	0.47

**Fig. 4.**
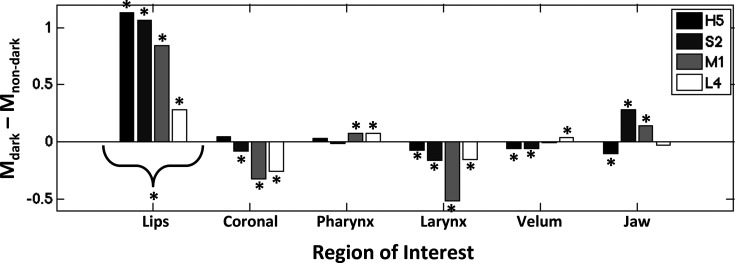
Mean formant frequency (F) differences between the dark and non-dark performance conditions, expressed as a proportion of mean formant frequencies during the normal performance condition. Differences are shown for each formant, 1–3, with bars representing subject-specific differences. Negative difference values correspond to lower mean formant frequencies during the dark performance. Asterisks (*) indicate corresponding significant effects, described in Sec. 3, within subjects (below bars) and across subjects (curly brackets).

Mean differences in formant frequencies, comparing dark and non-dark performance conditions, can be seen in Fig. 4 for each subject. All sopranos exhibited within-subject significant differences in mean F_1_ and F_2_ (*p* ≪ 0.001). Both M1 and S2 exhibited within-subject differences in mean F_3_ that were well below the significance threshold (both M1 and S2, *p* ≪ 0.001), while L4 exhibited differences in mean F_3_ that were slightly below the threshold (*p* = 0.04) and H5 did not show a significant difference in mean F_3_ (*p* = 0.75). Across subjects, there was a significant difference in mean F_1_ [*t*(3) = –4.055, *p* = 0.014] and mean F_2_ [*t*(3) = –3.074, *p* = 0.028], while the difference in mean F_3_ was somewhat above the threshold for significance [*t*(3) = –1.872, *p* = 0.079].

## 4. Discussion

In looking for aspects of vocal tract shaping associated with dark tone quality, the only such aspect that was consistent and statistically significant across all sopranos in the data set was the narrowing of the labial region. Substantial widening of the coronal region and the larynx were also observed across individuals, with the notable exception of H5's slight coronal narrowing. Though not statistically significant across sopranos, it is notable that the magnitude associated with differences in the coronal and laryngeal regions was substantially larger across subjects than those associated with the pharynx, velum, and jaw, which may suggest a role for coronal and laryngeal widening and which may represent a real effect that would achieve statistical significance given a larger number of study participants.^2^ Differences across subjects are not consistent or statistically significant with regard to the pharynx, velum, and jaw.

When looking at individual subjects, clear differences in vocal tract shaping can be observed when comparing dark and non-dark tone qualities in terms of the average position of all regions considered. All sopranos individually made use of labial narrowing in their performance involving dark tone quality, a trend that was also statistically significant across sopranos, as mentioned before. Beyond that, however, many significant differences were observed, but with abundant individual variability. H5 exhibited a widening of the larynx, accompanied by the lowering of the velum and the jaw. S2 also exhibited a widening of the larynx and a lowering of the velum but tended to raise the jaw and also widen the coronal region. M1 also displayed a widening of the coronal region, along with a narrowing of the pharynx, and a widening of the larynx, all of which was accompanied by a raising of the jaw. L4 also displayed a widening of the coronal region and a narrowing of the pharynx, as well as a widening of the larynx, but tended to raise the velum.

The acoustic analysis presented here provides further evidence that formant lowering (particularly of F_1_ and F_2_) is a key acoustic correlate of dark tone quality. However, the articulatory results shed new light on the production of dark tone quality, in that they differ in important ways from traditional accounts, which emphasize the importance of pharyngeal widening. Although the pharynx does seem to play a role, that role is highly variable across individuals, perhaps reflecting individual differences in training or inherent differences in individual anatomy, morphology, or biomechanics of the vocal articulators, which has been shown to influence vowel production (e.g., Lammert *et al.*, 2013a). The present results suggest that labial aperture may be the primary and most consistent driver of dark tone quality, which may also be related to earlier suggestions that labial widening is important for the production of “twang” (Story *et al.*, 2001).

The importance of labial narrowing shown in the present work further reinforces the idea, supported by recent work on vocal tract shaping during singing, that shaping of the lips—both narrowing and widening—plays a central role in many aspects of vocal performance. Echternach and colleagues have used MRI data in several studies to show that labial widening is associated with increases in loudness and pitch (Echternach *et al.*, 2016), with achieving modal register and belting styles (Echternach *et al.*, 2014a), and with supporting modal register at high pitch in tenors (i.e., above the *passaggio*). A study by Saldias *et al.* (2019) also indicates a role for wider lip opening in belting style. Titze and Worley (2009) and Titze *et al.* (2011) have used MRI data and physical acoustic simulations to explain how vocal performers might use vocal tract shaping to manage interactions between the vocal source and vocal tract resonances, perhaps with the goal of manipulating formant frequencies to maximize the acoustic energy generated in the larynx and the stability of vocal fold vibrations. They demonstrate that the “inverted megaphone” shape, necessarily featuring narrowing at the lips, is one way of effectively managing these interactions. The present work offers an additional explanation for why vocal performers might look to narrow the lips during some kinds of vocal performance, specifically to achieve a darker tone quality. It is not clear, however, how singers manage to navigate the apparently complex and often conflicting landscape of acoustic goals and their articulatory underpinnings. This challenging interplay is noteworthy and merits further study.

From a certain perspective, it is perhaps unsurprising that vocal performers would utilize narrowing of the lips when hoping to achieve a dark tone quality. That narrowing the lips results in a reduction of all formant frequencies has become a textbook example in the domain of phonetics (Ladefoged and Johnson, 2014; Johnson, 2004) and is predictable on the basis of the physics of resonant tubes (Fant, 1960; Mrayati, 1988; Story, 2005). One such prediction can be made based on the expected standing wave patterns in the vocal tract and the profile of volume velocity along its length associated with vibratory modes. Following perturbation theory (e.g., Stevens, 1998), narrow constrictions made near antinodes of a volume velocity profile will tend to decrease the resonant frequency of the corresponding mode. Owing to the fact that the volume velocity profile of each mode has an antinode near the lips, a narrowing at the lips is expected to decrease the frequency of all resonances. It is worth noting that performers have other methods available to them for decreasing all formant frequencies. An equally straightforward method predicted by perturbation theory would be to widen the larynx immediately superior to the glottis, which is expected to decrease the frequency of all resonances because the volume velocity profile of each mode has a node in that section of the vocal tract. The present results provide some evidence that individual sopranos may utilize this method of lowering formant frequencies (especially subject M1), even if no significant across-subject effect was found in this regard. Additionally, the physics of resonant tubes would also imply that lengthening the vocal tract has a similar formant-lowering effect, which could be achieved through protrusion of the lips. Further analysis would be required to determine if protrusion is used by performers to lower formant frequencies in pursuit of dark tone quality, perhaps in conjunction with labial narrowing in a rounding (in the phonetic sense) action of the lips overall.

It should be noted that several studies have shown a small but measurable influence of assuming a supine position on vocal tract shaping in the context of singing and during speech production, which might also exert a similar influence during singing. Traser *et al.* (2013) found a tendency for vocal performers to display a higher larynx position and a more protruded jaw while in supine position, but the overall effect was considered to be small. Kitamura *et al.* (2005) showed a tendency for speakers to adopt a slightly posterior tongue position in supine posture, while Stone *et al.* (2007) showed individual differences in adopting tongue positions that were more anterior or posterior in supine position. Shiller *et al.* (1999) found that speaker jaw positions and formant frequencies were somewhat different while in supine posture, and Hoedl (2015) found related acoustic differences. Most studies show effects that are highly subject-specific. Efforts to characterize vocal tract shaping in dark tone quality using these data may be influenced by effects such as those mentioned in these studies. However, these effects would not be expected to systematically vary with performance style conditions in the present study and therefore would not be expected to confound the present results.

Future work will expand the current analysis to additional regions of the vocal tract (e.g., off the midsagittal plane) and additional details of tone quality production kinematics. It is likely that the chosen ROIs do not capture all articulatory variance present in the image sequences. Given the detailed information about vocal tract shaping captured by rtMRI, an exploratory analysis of articulatory changes may be merited as a complement to the present theoretically driven approach. Future work will also consider the possibility of interactions between phonemic context and production of tone quality. Prior studies have indicated that aspects of vocal tract shaping during vocal performance, particularly those related to maintaining register (Echternach *et al.*, 2014b) and managing source-filter interactions (Titze *et al.*, 2011), are vowel-dependent. The present analysis, which looks at overall, average differences in vocal tract shaping, is limited in its ability to identify such vowel-specific effects, although such effects may be merited as the focus of future analyses. Finally, the impact of tone quality production on vocal health will also be considered in future work, with potential applications to improved training and vocal pedagogy.

## Footnotes

^1^ See supplementary material at https://www.scitation.org/doi/suppl/10.1121/10.0005109 for details of the physical acoustic analysis.

^2^ These patterns of coronal and laryngeal widening were also predicted by the physical acoustic simulations, presented in the supplementary material, further reinforcing their potential importance in dark tone quality.
